# Feasibility and outcomes of cytoreductive surgery and HIPEC for peritoneal surface malignancies in low- and middle-income countries: a single-center experience of 232 cases

**DOI:** 10.1186/s12957-021-02276-5

**Published:** 2021-06-05

**Authors:** Suryanarayana Deo, Mukurdipi Ray, Babul Bansal, Sandeep Bhoriwal, Sushma Bhatnagar, Rakesh Garg, Nishkarsh Gupta, Atul Sharma, Lalit Kumar, Sanjay Thulkar, Ekta Dhamija, Sandeep Mathur, Prasenjit Das

**Affiliations:** 1 Department of Surgical Oncology, Dr. BRA-IRCH, AIIMS, New Delhi, 110029 India; 2 Department of Onco-anesthesia & Palliative Medicine, BRA-IRCH, AIIMS, New Delhi, India; 3 Department of Medical Oncology, BRA-IRCH, AIIMS, New Delhi, India; 4 Department of Radiodiagnosis, BRA-IRCH, AIIMS, New Delhi, India; 5 Department of Pathology, AIIMS, New Delhi, India

**Keywords:** Peritoneal surface malignancy, Cytoreductive surgery, HIPEC, LMIC, Tertiary care center

## Abstract

**Background:**

Cytoreductive surgery (CRS) and hyperthermic intraperitoneal chemotherapy (HIPEC) has recently emerged as a viable management option for peritoneal surface malignancy (PSM). CRS and HIPEC is a complex, multidisciplinary and resource-intensive surgical procedure. It has a steep learning curve and is associated with significant morbidity and mortality. The expertise is mostly limited to few dedicated high-volume centers located in developed countries. We present a single institutional experience of 232 cases of CRS and HIPEC performed at a tertiary care cancer center in a low- and middle-income country (LMIC).

**Methods:**

A multidisciplinary PSM program was initiated in 2015 at a high-volume public-sector tertiary care cancer center in North India catering largely to patients belonging to low- and middle-income groups. Perioperative protocols were developed, and a prospective structured database was created to capture data. All patients undergoing CRS and HIPEC between January 2015 and December 2020 were identified, and the data was retrospectively analyzed for clinical spectrum, surgical details, and perioperative morbidity and mortality.

**Results:**

Two hundred and thirty-two patients underwent CRS and HIPEC during the study period. Epithelial ovarian carcinoma (56.5%) was the most common malignancy treated, followed by pseudomyxoma peritonei (18.5%), colorectal carcinoma (13.4%), and malignant mesothelioma (5.6%). Optimal CRS could be achieved in 94.4% of patients. Cisplatin and mitomycin were the most common drugs used for HIPEC. A total of 28.0% of patients had morbidity including deep vein thrombosis, subacute intestinal obstruction, sepsis, burst abdomen, lymphocele, urinoma, acute renal failure, and enterocutaneous fistula. The overall treatment-related mortality was 3.5%.

**Conclusions:**

Results of the current study indicate that it is feasible to establish a successful CRS and HIPEC program for PSM in government-funded hospitals in LMIC facing resource constraints. The most common indication for CRS and HIPEC were carcinoma of the ovary followed by pseudomyxoma peritonei and colorectal carcinoma. Overall morbidity and mortality in the current series are comparable to global standards, reported from high-income countries. A protocol-based multidisciplinary team approach, optimal patient selection, and surgical expertise can help achieve optimal outcomes in government-funded hospitals in LMIC.

## Background

Peritoneal surface malignancies (PSM) comprises of a group of neoplasms which either disseminate through or arise from peritoneal membrane [1]. Till recently, patients suffering with PSM were considered incurable, due to lack of effective therapy and limited survival. The therapeutic approach to PSM was nihilistic and involved attempts at debulking surgery and delivering palliative chemotherapy. However, over the last two decades, significant advancements have been made in the field of PSM. Cytoreductive surgery (CRS) in combination with hyperthermic intraperitoneal chemotherapy (HIPEC) has emerged as a promising intervention in a subset of PSM patients [2].

Only a few centers have been able to achieve proficiency in CRS and HIPEC as it is a complex, multidisciplinary and resource-intensive intervention. Initial experience of CRS and HIPEC was marred with high morbidity and mortality and a significant learning curve [3]. In the recent past, an increasing number of centers globally have initiated successful CRS and HIPEC programs, largely due to standardization of patient selection criteria, surgical technique, and perioperative protocols. To consolidate the evidence base, a large number of prospective randomized trials are currently being conducted by multiple centers.

Most of the literature related to CRS and HIPEC has been published by centers from high-income countries (HIC). There is paucity of literature from low- and middle-income countries (LMIC) despite having a significant volume of patients with PSM. The challenges in establishing complex and expensive treatment modality like CRS and HIPEC in LMIC include lack of expertise, resource constraints, high patient volumes, and socioeconomic factors.

In this article, we aim to present our experience of establishing a PSM program at a government-funded tertiary care cancer center in LMIC utilizing pre-existing resources and present outcomes of treatment in 232 PSM patients treated with CRS and HIPEC.

## Methods

The data was collected from a prospectively maintained computerized database of the department of surgical oncology based in a government-funded tertiary care comprehensive cancer center in northern part of India. All patients who had undergone CRS and HIPEC between January 2015 and December 2020 were included in the study. Records of these patients were reviewed for details regarding the clinical spectrum, surgical details, perioperative (i.e., till 30 days after surgery) outcomes including morbidity and mortality. A retrospective analysis was performed.

### Details of program and protocol development

The team for management of peritoneal surface malignancies at our center is multidisciplinary in nature. The team includes experienced doctors from the departments of surgical oncology, medical oncology, radiodiagnosis, and pathology. The core CRS and HIPEC team comprises of experienced senior surgical oncologists, dedicated onco-anesthesia & critical care experts, trained nursing and operation theatre technicians. The support team helps in prehabilitation and postoperative care of the patient and includes members from physiotherapy, nutrition, stoma care, and clinical psychology divisions. Initially, all the procedures were performed by two lead senior surgical oncologists experienced in performing complex surgical oncology procedures and re-do abdominal surgeries after attending dedicated CRS and HIPEC workshops/courses and short-term observerships in high-volume PSM centers. Subsequently, trainees were also allowed to perform simple peritonectomies under supervision.

The prehabilitation protocol includes nutritional status assessment using Subjective Global Assessment (SGA) followed by intensive nutritional support in patients with average or poor nutritional status (viz. body mass index less than 18.5, weight loss more than 10% over last 6 months, serum albumin less than 3 g/dL). Assessment of the baseline physical status is done by a “6-min walk test,” pulmonary function test (PFT), and cardiopulmonary exercise testing (CPET). Respiratory conditioning is done by the physiotherapy team with incentive spirometry and a program of home exercises. Cardiovascular status assessment and optimization is done by the anesthesiology and cardiology team in patients with such comorbidities.

Standard patient selection criteria based on guidelines available are followed [4]. PSM patients with a good performance status (ECOG PS ≤ 1) and no or well-controlled comorbidities are offered CRS and HIPEC. Selected patients with ECOG PS 2 are also offered CRS and HIPEC after prehabilitation. Patients with unfavorable tumor biology (e.g., signet ring cell adenocarcinomas, sarcomatoid variant of malignant mesothelioma), high CT-PCI (i.e., greater than 20), or features on imaging deemed unsuitable for optimal cytoreduction are excluded [5]. CT-PCI is calculated by locating tumor deposits in the 13 regions of the abdomen as described by Koh et al. [6].

The decision to administer neoadjuvant chemotherapy and regimen is taken in the multidisciplinary tumor board meetings. Advanced ovarian cancer patients with high clinical tumor burden are offered neoadjuvant chemotherapy (NACT) followed by interval CRS and HIPEC. Epithelial ovarian cancer patients receive 3 to 6 cycles of standard platinum-based chemotherapy, if platinum naïve or platinum sensitive. Otherwise, they receive second line chemotherapy. Colorectal cancer patients with peritoneal metastases received NACT using standard FOLFOX regime [7]. Meticulous exploratory laparotomy is performed for calculation of PCI and predicting feasibility of optimal CRS. The goal of CRS is to achieve optimal cytoreduction which is defined as no macroscopic residual disease (CC-0) or macroscopic disease nodules ≤ 2.5 mm in size (CC-1). Larger residual macroscopic disease (CC-2/CC-3) is considered suboptimal cytoreduction [8]. Total or disease-specific partial peritonectomy is performed to achieve optimal cytoreduction at the discretion of the operating surgeon [9, 10]. Total peritonectomy comprises removal of parietal peritoneum from bilateral hemidiaphragm, anterior abdominal wall, pelvis, paracolic gutter and diaphragmatic recesses, and greater and lesser omentum along with partial visceral peritonectomy as described by Sugarbaker et al. Multivisceral resections are performed when indicated to achieve optimal CRS status.

**Fig. 1:**
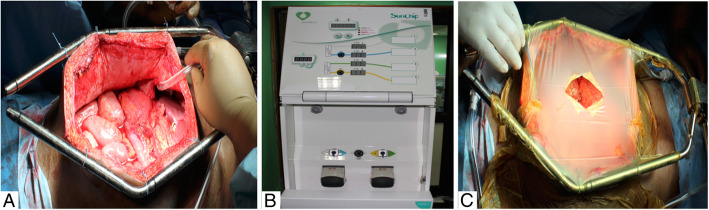
HIPEC technique. **a** Suspension of abdominal wall edges to Omnitract^TM^ retractor to create a Coliseum. **b** Sunchip^TM^ machine. **c** Coliseum covered with Steri-Drape^TM^ (semi-open)

After completion of cytoreductive surgery, HIPEC is instituted using a Sunchip^TM^ machine (manufactured by GamidaTech® France). A semi-open technique is used with the help of Omnitract^TM^/Thompson^TM^ retractor and suspension of abdominal wall edges. A Steri-Drape^TM^ was used to temporarily cover the laparotomy wound with a central opening to ensure adequate filling of perfusate and for agitation of abdominal contents for uniform distribution of heated perfusate (Fig. 1).

Choice of chemotherapeutic agent for intraperitoneal chemotherapy is based on the standard recommendations. Generally, cisplatin is used for platinum-sensitive ovarian tumors and mitomycin-C for non-platinum-sensitive ovarian malignancies and gastrointestinal malignancies. The choice of drug and dose is decided by the multidisciplinary oncology team. Normal saline is used as carrier solution, and HIPEC is instituted for 45 to 60 min at a temperature range of 41 to 42°C. Standard safety precautions are followed including prevention of spillage, smoke evacuation, and personnel protective equipment. Intraoperatively, standard protocols are followed by anesthesia team pertaining to maintenance of core temperature, hemodynamic stability, infusion of IV fluids/blood products, and maintaining adequate urine output. Postoperatively, patients are monitored in ICU for 24 to 48 h.

The perioperative morbidities related to CRS and HIPEC are documented meticulously in a predesigned pro forma. After discharge, patients are reviewed again by the multidisciplinary team to assess the need for adjuvant therapy based on primary tumor type, surgical outcome, and final histopathology report.

The cost of the CRS and HIPEC treatment is subsidized by the hospital, and patients are charged for disposable HIPEC sets making the treatment affordable to patients belonging to low and middle socioeconomic strata.

## Results

**Fig. 2:**
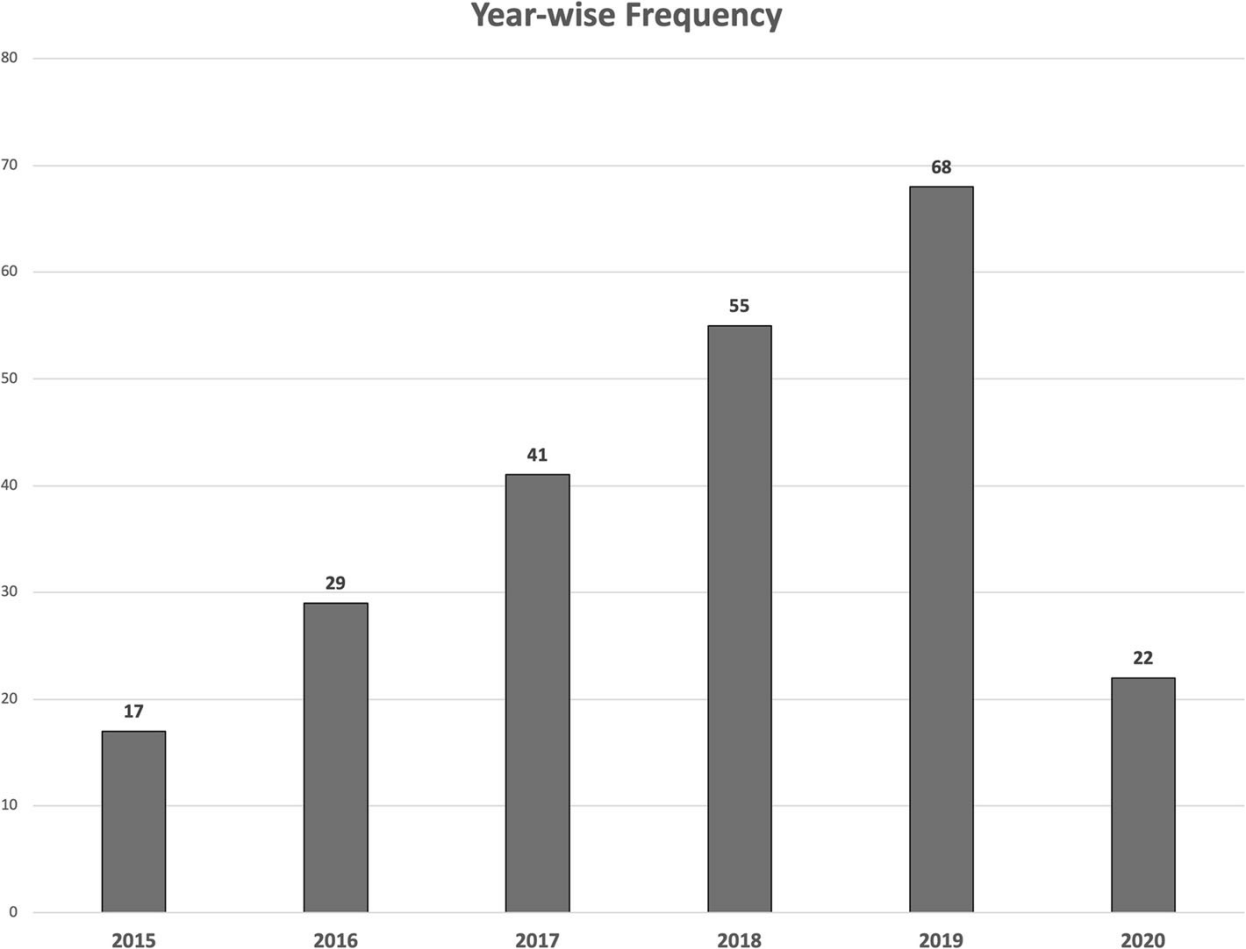
Year-wise trends of CRS and HIPEC

A total of 232 patients with PSM underwent CRS and HIPEC between January 2015 and December 2020. The year-wise frequency of cases is shown in Fig. 2. Most of the protocols were standardized during the first year, and subsequently, minor modifications were made. There was a steady increase in the volume of CRS and HIPEC cases during the study period. However, only a limited number of cases were performed in the year 2020, owing to the pandemic.

The details of clinical profile of patients are shown in Table 1. The mean age was 47.3 years, and there was female preponderance. Nearly one-third of patients (34.9%) had comorbidities, predominantly diabetes and hypertension.

**Table 1:** Demography and patient profile

Age [mean ± SD]	47.3 ± 9.2 years	
**Sex**	Male	36 (15.5%)
	Female	196 (84.5%)
**Performance status (ECOG)**	PS 0/1	214 (92.2%)
	PS 2	18 (7.8%)
	PS 3/4	0%
**Comorbidities**	None	151 (65.1%)
	Comorbidity	
	• Diabetes mellitus • Hypertension • Hypothyroidism • Coronary artery disease • COPD	51 (21.9%) 33 (14.2%) 12 (5.2%) 5 (2.2%)
		3 (1.3%)

Table 2 shows spectrum of PSM patients undergoing CRS and HIPEC. Ovarian carcinoma, pseudomyxoma peritonei, and colorectal cancers comprised 88.4% of cases in the current series. Rare and uncommon indications comprised 11.6% of cases.

**Table 2:** Clinical indications for CRS and HIPEC

Clinical indication	Number	Percentage
Carcinoma of the ovary	131	56.5%
Pseudomyxoma peritonei	43	18.5%
Colorectal cancer	31	13.4%
Malignant mesothelioma	13	5.6%
Gastric carcinoma	6	2.6%
Endometrial stromal sarcoma	5	2.2%
Granulosa cell tumor	3	1.3%

Table 3 shows surgical details of patients undergoing CRS and HIPEC. Mean operating time was 379±108.7 min. The mean peritoneal carcinomatosis index (PCI) was 9.3 with a range of 3 to 26. The mean PCI of non-ovarian cancer group was higher than ovarian cancer group due to usage of neoadjuvant chemotherapy (NACT) leading to downstaging in majority of ovarian cancer patients. 84.7% of ovarian and 40.6% of non-ovarian cancer patients underwent NACT. Total peritonectomy was performed in 21.1% of cases, and this decision was based on disease type and extent of peritoneal involvement. 94.4% of patients had an optimal CRS (CC-0/CC-1) before institution of HIPEC. Multivisceral resection (i.e., resection of viscera other than the primary site of malignancy) was performed in 24.5% of patients. Cisplatin was the most common drug used for HIPEC. The patients spent an average of 1.3 days in ICU and 8.1 days in hospital postoperatively.

**Table 3:** Details of CRS and HIPEC

Operative time [mean ± SD]	379.9 ± 108.7 min	
**PCI** [mean (range)]	Total (n=232)	9.3 (0–26)
	- Non-ovarian group (n=101) - Ovarian group (n=131)	- 11.1 (0–25) - 7.9 (0–26)
**Extent of peritonectomy**	Total peritonectomy Disease-specific peritonectomy	49 (21.1%) 183 (78.9%)
**Types of CRS**	Optimal CRS (CC-0/CC-1) Suboptimal CRS (CC-2/CC-3)	219 (94.4%) 13 (5.6%)
**Multivisceral resections**	Hysterectomy/salpingo-oophorectomy Appendicectomy Colectomy Anterior/low-anterior resection Small intestine -- resection/anastomosis Splenectomy +/- distal pancreatectomy Cholecystectomy Urinary bladder–partial resection	42 (18.1%) 38 (16.4%) 22 (9.5%) 3 (1.3%) 21 (9.1%) 8 (3.5%) 16 (6.9%) 4 (1.7%)
	Total	57 (24.5%)
**Drugs for HIPEC**	Cisplatin Mitomycin C Oxaliplatin Adriamycin Melphalan	191 (82.3%) 27 (11.6%) 7 (3.0%) 5 (2.2%) 2 (0.9%)
**ICU stay**	1.3 (1–12) days	
**Postoperative hospital stay**	8.1 (5–26) days	

Table 4 shows details of perioperative morbidity (NCI-CTCAE v5.0 Gr. 3-4) which occurred in 28.0% of patients. The most common major morbidity was deep venous thrombosis followed by subacute intestinal obstruction (SAIO), sepsis, burst abdomen, lymphocele, neutropenia, urinoma, acute renal failure, and enterocutaneous fistula

**Table 4:** Spectrum and incidence of perioperative morbidity

Morbidity	Number of patients	Percentage
Deep venous thrombosis	15	6.5%
Subacute intestinal obstruction	14	6.0%
Sepsis	12	5.2%
Burst abdomen	10	4.3%
Neutropenia	9	3.9%
Lymphocele	9	3.9%
Urinoma	7	3.0%
Acute renal failure	4	1.7%
Anastomotic leak	4	1.7%
Pulmonary embolism	3	1.3%
Acute respiratory distress syndrome (ARDS)	2	0.9%
GTCS + cardiac arrest (revived)	1	0.4%
Biliary peritonitis	1	0.4%
**Overall**	65	28.0%

Perioperative mortality occurred in 8 cases (3.5%). Four patients died due to sepsis leading to acute respiratory distress syndrome (ARDS), 2 patients had sudden death on the first postoperative day due to suspected pulmonary embolism, 1 patient succumbed to biliary peritonitis, and 1 patient due to cardiac arrest in postoperative period.

## Discussion

Over the last two decades, with the advances made in the field of CRS and HIPEC, peritoneal surface dissemination of abdominal malignancies is increasingly being recognized as a regional disease amenable to potential cure, in a subset of patients. One of the major breakthroughs that helped in mainstreaming CRS is the surgical standardization of total peritonectomy by Paul H. Sugarbaker in 1995 [9, 11]. Parallel advancements in the field of HIPEC have resulted in increased utilization of CRS and HIPEC as an effective combination therapy for PSM by a number of centers [2]. Last decade has witnessed a spurt in publication of literature pertaining to CRS and HIPEC mainly from HIC. Due to the complexity of treatment, cost factors, and associated higher morbidity and mortality, very few centers from LMIC have ventured in to establish CRS and HIPEC programs despite having a significant volume of patients with PSM. There is a need to share the experience of establishing a CRS and HIPEC program from resource-constrained settings to assess the feasibility, safety, and efficacy.

India has a mixed healthcare system, inclusive of public and private health-care service providers [12]. The public healthcare service is a three-tiered system, providing primary, secondary, and tertiary levels of care. These services are funded by the government to a large extent. However, being a LMIC with a burgeoning population of 1.36 billion, Indian governments are able to dedicate only 1% of the GDP to public healthcare services. Only 16% of the population is covered by some form of health insurance [13]. The average per capita income in India is 150 USD per month, and availing private sector health care facility is beyond the reach of most [14]. This places an undue burden on tertiary health care facilities in the public domain, and starting a new program by using the already strained resources is a difficult proposition.

In this article, we present the experience of first 232 cases of CRS and HIPEC at our center. Our hospital is the apex tertiary care hospital in northern part of India, being funded by the government of India. We have established a peritoneal surface malignancy program using the existing infrastructure and man power in our department. The economic model used for this program was mixed in nature. The HIPEC machine was procured using funds from the government of India, and the services were provided free of cost. The patients were only required to buy the consumables used in the operation of HIPEC machine (approximate cost–USD 1200). The patients with income below poverty line were even provided the consumables free of cost using the existing government welfare schemes.

CRS and HIPEC is a time- and resource-intensive intervention, and for initiation of long-term viable CRS and HIPEC program, a good pre-existing healthcare infrastructure and a multidisciplinary team approach is essential. Other important issue is optimal patient selection for CRS and HIPEC. These procedures should only be offered to the group of patients who are likely to benefit, so as to optimize resource utilization. Two factors need to be considered while selecting patients for CRS and HIPEC. First is the disease type, and the other is the extent and volume of peritoneal spread. Based on current literature pseudomyxoma peritonei, mesothelioma, peritoneal metastases of tumors of appendicular and colorectal origin, and ovarian cancers have shown benefit with CRS and HIPEC [15–22]. CT scan of the abdomen and pelvis is used to assess volume and extent of peritoneal spread and helps to determine feasibility of optimal cytoreduction. Performance status of the patient and optimization of comorbidities are key factors in patient selection process. We followed stringent patient selection criteria in the current study. Two thirds of the patients had no comorbidity, and the remaining had co-existing diseases like diabetes mellitus, hypertension, and hypothyroidism which were well controlled. Patients with aggressive histologies (e.g., signet ring cell adenocarcinoma) were not selected for these procedures.

Spectrum of patients undergoing CRS and HIPEC varies in different studies. Factors which can influence the disease spectrum include type of surgical specialty and referral patterns. Most of the studies evaluating CRS and HIPEC from the west have a preponderance of tumors of gastrointestinal tract origin. In a study published by Levine et al. comprising 1000 patients, 47.2% of the tumors were of appendiceal origin and 24.8% colorectal origin, and only 6.9% were that of ovarian origin [23–25]. In the current study, more than half of the patients had tumors of ovarian origin. Peritoneal metastases of colorectal origin and pseudomyxoma peritonei comprised one fourth of the volume. These trends are likely to change over a time period due to referral patterns and revision of guidelines based on results from ongoing randomized studies.

The average peritoneal carcinomatosis index (PCI) in our series was 9.3, lower than that reported in other contemporary series where mean PCI ranged from 11 to 16 [23, 26–28]. Perhaps, this is because of a stringent selection criteria used and preponderance of cases of carcinoma ovary receiving prior chemotherapy resulting in down staging and low PCI. The mean PCI for non-ovarian cancer patients was 11.1 in our series.

In view of the complexity and variations in protocols reported in literature, every institution planning to start CRS and HIPEC programs should have standard and uniform protocols pertaining to patient selection, prehabilitation, surgical technique, HIPEC methods, anesthesia, and ICU care for consistent outcomes. Meticulous documentation of patient and treatment details preferably in a structured electronic format is important, as it helps in performing audits for learning and facilitates analysis of data for outcomes .

Preliminary meticulous exploration of the abdomen to document PCI and assess feasibility of optimal CRS is an important step which helps in early decision-making. We suggest a senior and experienced surgeon be involved in this part of the procedure. It is recommended to have a team of surgeons, well versed with the intricacies of CRS, to work together to avoid fatigue and errors. At our institution, two experienced surgeons performed majority of the procedures during the initial phase, and subsequently, trainees were allowed to perform simple peritonectomies under supervision. The surgical goal of CRS is to achieve an optimal cytoreduction status, balancing risk versus benefit. In the current study, 21.1% had total peritonectomy, and the remaining had partial disease-specific peritonectomy. For the HIPEC phase of surgery, adherence to safety protocols using safe and efficacious cytotoxic agents is key to success. Dosages of prior chemotherapy a patient has received should always be kept in mind while deciding the intraperitoneal chemotherapy dose so as to avoid toxicity.

Most of the initial studies reported very high morbidity and mortality with CRS and HIPEC [29, 30]. However, with increasing experience, contemporary studies are reporting an acceptable rate of morbidity and mortality [24, 31–33]. In the current study, perioperative morbidity occurred in 28.0% of patients. The most common morbidities encountered were deep vein thrombosis, subacute intestinal obstruction, sepsis, and burst abdomen. Morbidity of the current study is comparable to the morbidity reported in recent studies. Overall mortality in our experience was 3.5% which was acceptable in comparison to mortalities reported in recent studies ranging from 2 to 8.6%.

## Conclusion

In conclusion, outcomes of the current study indicate that it is feasible to implement complex and resource-intensive treatment like CRS and HIPEC in resource-constrained hospitals in LMIC. The key to success is multidisciplinary team approach, protocol-based treatment delivery with strict adherence to patient selection criteria, surgical quality control, and optimal perioperative care.

## Data Availability

The data was extracted from the computerized database in the Department of Surgical Oncology at AIIMS, New Delhi, and will be available from corresponding author on reasonable request.

## Abbreviations

CRS: Cytoreductive surgery
HIPEC: Hyperthermic intraperitoneal chemotherapy
PSM: Peritoneal surface malignancy
LMIC: Low- and middle-income country
HIC: High-income country
SGA: Subjective Global Assessment
PFT: Pulmonary function test
CPET: Cardiopulmonary exercise testing
ECOG: Eastern Cooperative Oncology Group
PS: Performance status
NACT: Neoadjuvant chemotherapy
CC: Completeness of cytoreduction
IV: Intravenous
ICU: Intensive care unit
CT: Computed tomography
PCI: Peritoneal carcinomatosis index
NCI-CTCAE: National Cancer Institute–Common Terminology Criteria for Adverse Events
Gr.: Grade
SAIO: Subacute intestinal obstruction
ARDS: Acute respiratory distress syndrome
